# Design of Protein Segments and Peptides for Binding to Protein Targets

**DOI:** 10.34133/2022/9783197

**Published:** 2022-04-15

**Authors:** Suchetana Gupta, Noora Azadvari, Parisa Hosseinzadeh

**Affiliations:** Knight Campus Center for Accelerating Scientific Impact, University of Oregon, Eugene OR 97403, USA

## Abstract

Recent years have witnessed a rise in methods for accurate prediction of structure and design of novel functional proteins. Design of functional protein fragments and peptides occupy a small, albeit unique, space within the general field of protein design. While the smaller size of these peptides allows for more exhaustive computational methods, flexibility in their structure and sparsity of data compared to proteins, as well as presence of noncanonical building blocks, add additional challenges to their design. This review summarizes the current advances in the design of protein fragments and peptides for binding to targets and discusses the challenges in the field, with an eye toward future directions.

## 1. Introduction

**Figure 1:**
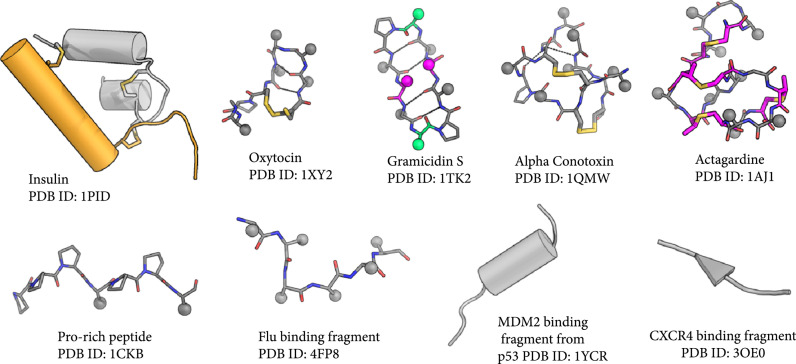
Peptides can take on different structures. Structures of several natural peptides are shown. Sticks show crosslinks. Magenta color highlights noncanonical AAs. Green residues are D-AAs. The spheres show the position of sidechain C-*β* atoms. Dashed lines represent hydrogen bonds.

Peptides, short stretches of amino acids (AAs), often smaller than 50 residues, play key roles in our cellular function (Figure 1, Table 1). Many of these peptides act as hormones that transfer messages in our body; metabolic hormones such as insulin and neuropeptides such as oxytocin are two examples of peptide hormones. Some protein fragments (also called peptides, hereafter) are in charge of modulating our cell signaling cascades such as signal peptides that guide proteins to their proper cellular locations, or proline-rich peptides that interact with SH3 domains in multiple signaling pathways. Some peptides play a role in defense; antimicrobial peptides (AMPs) and many antibiotics such as gramicidin S and lantibiotics and some toxins represent this class. This diversity in function suggests that designing peptides can open the door for many applications, from tuning cell signaling to generating novel antibiotics.

**Table 1:** Description of different classes of peptides, their descriptions, and some of their general features, and examples of them with structure.

Type of peptide	Subclasses	General features	Example
Cyclic peptides Peptides with the N- and C-termini connected via a bond (N-to-C amide bond, disulfide bond, thioether bond, …)	Unicyclic	Can be chemically synthesized, can include ncAAs, often <20 AA, often lack clear secondary structure elements, often protease resistant	Cyclosporin (11mer N-to-C cyclized peptide) binds to cyclophilin (PDB 1CWA [1])
	Multicyclic: where there are additional internal bridges such as disulfide bonds in a cyclic peptide		G7-B1 (bicyclic 11mer) binds to Grb7-SH2 domain (PDB 5EEL [2])

Lariat peptides Cyclic peptides with linear segments flanking from one or both termini	Lariats	Can be chemically synthesized, can include ncAAs	Oxytocin binds to oxytocin receptor (NMR structure of unbound oxytocin: 2MGO [3])

Lasso peptides A ring with a C-terminal tail that goes through the ring [4]	Class I: 2 disulfide bonds between the tail and the ring	A member of ribosomally assembled posttranslationally modified peptides (RiPPs), often hard to synthesize, heat resistant	RP 71955 (PDB 1RPC [5] shows structure in solution)
	Class II: no disulfide bond		Caulosegnin I (PDB 2LX6 [6] shows structure in solution)
	Class III: one disulfide bond between the tail and the ring		BI-32169 (PDB 3NJW [7] shows structure in solution)

Constrained peptides Peptides that are stabilized by multiple internal cross-linking bridges	Disulfide constrained peptides such as conotoxins	Very stable, can be chemically synthesized or expressed, often resistant to loop swapping	A-conotoxin binds to nicotinic acetylcholine receptor (PDB 2C9T [8] in complex with AChBP homolog)
	Peptides with multiple noncanonical crosslinkers such as lantibiotics	Often naturally synthesized and posttranslationally modified, often include a large variety of	Nisin (PDB 1WCO [9] shows Nisin in complex with lipid-II)

Linear fragments	Helical fragments	Can be expressed or synthesized, originally no ncAAs, often require additional crosslinkers or scaffolds to stabilize the given conformation	Helical fragment of p53 bound to MDM2 (PDB 1YCR [10])
	β-Strand fragments		C-terminal of p53 bound to SIRT1 (PDB 4ZZJ [11])
	Loop fragments		Proline rich peptides (PDB 1GBR [12])

Miniproteins	Can be classified based on the order of secondary structure elements, or presence or absence of disulfide bonds	Larger peptides often around 20-50 amino acids, stabilized by a packed core, can have disulfide bonds as well, often expressed but in the case of shorter ones can be synthesized	Miniprotein anti-ACE binder (PDB 7JZM [13])

In addition to their natural roles, peptides have been used as a therapeutic modality complementary to antibodies and small molecules [14–17]. Similar to antibodies, peptides can bind to flat protein surfaces with high affinities and selectivities. And similar to small molecules, they can cross the cell membrane to access intracellular targets. Thus, they offer a unique opportunity to target the so-called undruggable space of disease-related targets that are currently not accessible by antibodies or small molecules. While current methods for obtaining these therapeutic peptides often require a library-screening step, the ability to design peptides with desired properties to guide these libraries is of high interest.

Despite their important role both as natural molecules and as future therapeutics, design of functional peptides has been a challenging endeavor. In addition to challenges faced by the field of protein design in general, peptide design has its own unique limitations. One of the key difficulties in peptide design arises from the practical limitations in fully modeling the structural features of peptides. The abundance of noncanonical amino acids (ncAAs) in peptide sequences (Figure 1) necessitates additional parameterization of these amino acids for the force fields used for computational design. Additionally, these ncAAs confer unique structural features to the peptide that are not present in the large database of protein structures. Many peptides also include crosslinkers and/or are cyclized. The presence of these crosslinkers and cyclizations adds restraint to the backbone of the peptide and leads to peptide bonds or angles that deviate from the equilibrium seen in natural proteins. Unusual puckerings of the proline ring, and peptide bonds that are out of plane are examples of such deviations [18, 19]. Thus, the heuristic knowledge gained from studying protein structures is often not enough for modeling peptide structures, and learning methods trained on protein databases will not be generalizable to all peptides.

Another challenge in designing peptides is that the one-to-one structure-function link that is often very prominent in functional proteins is blurry for peptides. In fact, many functional peptides are intrinsically disordered or sample multiple conformations [20, 21]. This conformational plasticity complicates the design process in two ways. First, it adds complexity to the already challenging design process. Second, since these conformational ensembles are often harder to experimentally validate, the number of examples one can learn from to create novel peptides is more limited compared to stable proteins with one conformation. Finally, due to their small size, many peptides lack secondary structures that are the fundamental units of designing proteins [22].

Designing functional peptides that bind to protein surfaces creates an additional challenge to the design: correct modeling of the binding competent orientation. Sampling this conformation is often done via docking or molecular dynamics simulations. However, these methods often suffer from long run times and/or low accuracy. Part of this challenge is due to insufficient sampling and ranking of generated docked models. Another contributor is the difficulty in modeling water molecules at the interface. It is well known that many peptides bind to their protein targets via water-mediated hydrogen bonds [23]. However, despite current advances in computational modeling of water molecules [24], accurate modeling of water molecules at the interface is still a computationally challenging task.

**Figure 2:**
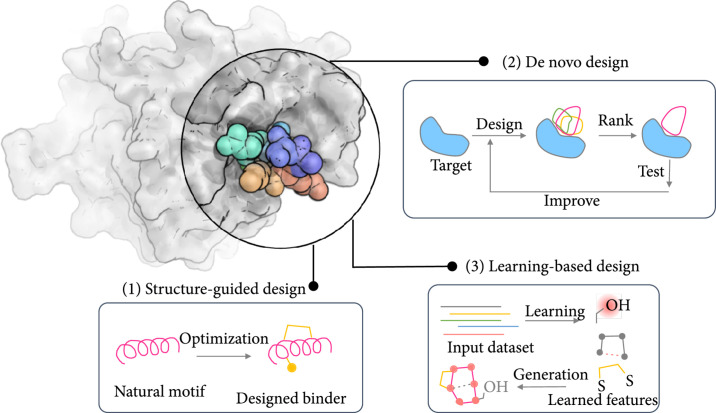
Schematic representation of different methods used for designing peptide binders. To obtain a peptide that binds to a surface of interest (shown in spheres within the circle in the top-left figure. Structure from PDB ID: 6WSJ), researchers can take on three general approaches: (1) structure-guided design, which uses natural motifs as a starting point to create the binders, (2) de novo design, which builds many potential peptide binders from scratch and ranks them to select the best candidates for testing, and (3) learning-based design, which takes advantage of machine learning and deep-learning methods to learn from available data and create peptide binders.

Despite these challenges, researchers have succeeded in designing peptides covering a wide range of functions: peptides that can bind to target proteins (topic of this review), catalytic peptides [25], and peptide-based higher-order assemblies [26, 27]. These methods can be broadly categorized into three strategies (Figure 2): (a) structure-guided, (b) de novo, and (c) learning-based methods. It should be noted that the lines between these methods are at times blurry and they are mainly used for structuring this review.

To provide a general overview of these design methods and the progress of the field, in the following sections we will focus the discussion on the strategies used for the design of peptides for their most common application, binding to targets, along with some representative examples.

## 2. Structure-Guided Design of Functional Peptides

One of the most successful strategies to design functional peptides is to start the design from an existing fragment (often ≥4 residues) or *motif*, that is known to have the function of interest, and improve upon that. These improvements are often minimal and retain the overall sequence of the peptide motif as well as its structure. Integrin-binding RGD loop is an example of such motifs [28]. We refer to these methods that are inspired by naturally occurring peptide motifs *structure-guided design*.

The motif is often extracted from natural proteins using available structures from PDB. The process of motif identification and extraction is slow and can be highly accelerated through the use of computational methods. For example, Tsai et al. [29] recently created TP-DB (therapeutic peptide design database) that allows the users to search helical motifs that match a given sequence pattern. Their pattern-specific search engine is based on features derived from nearly 1.7 million helical structures extracted from PDB. These identified helical motifs can provide a starting point for design of peptides starting from helical motifs. In another example, Alam et al. [30] used an algorithm called FlexPepBind as a screening tool in order to find potential substrates of histone deacetylase 8 (HDAC8). FlexPepBind uses the structural model of a peptide-protein complex to calculate the binding specificity of the peptide to its receptors. After calibrating the results of FlexPepBind over an existing set of peptides with known affinities for HDAC8, they successfully used FlexPepBind to fish out new substrates of HDAC8 by running it on all the peptides in human proteome that are reported to have acetylation sites. HADC8 showed reactivity towards 20 peptides from a set of 26 peptides that were experimentally tested.

**Figure 3:**
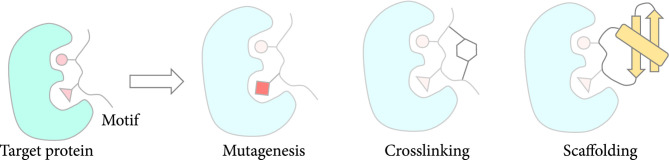
Structure-guided design methods start from a naturally occurring motif. The motif is then stabilized and improved upon through mutagenesis, crosslinking, or scaffolding. Often, a combination of methods is required to obtain peptides with desired affinities. Note that in some cases, a de novo designed peptide can be built around an already existing motif using some of these approaches (more details in Section 3).

After finding the motif, it is improved upon in one of several ways (Figure 3): (a) mutagenesis, (b) crosslinking, and (c) scaffolding. It is important to note that while motifs are important contributors to binding, in most cases the binding affinity of the protein partner from which the motif is extracted is better due to the contributions of interacting residues outside of the motif. Thus, as shown in some of the examples reviewed, in order to generate a peptide that can compete with the native binder, a combination of approaches is required. For example, a scaffolded motif will undergo a series of mutagenesis studies to generate binders that can compete with the native binder. These mutagenesis studies can be done in a high throughput way using library-based methods.

### 2.1. Designing Binders Using Motif Mutagenesis

Naturally occuring protein-protein interactions are evolved to perform a function. This function does not always correlate with high binding affinity. In fact, in many cases, promiscuous or low affinity transient binding events are required to carry out biological processes [31–33]. Thus, a known peptide-protein interaction can be strengthened by making mutations to the peptide at the binding interface (Table 2). Mutagenesis often starts with a change in sequence and then assessment of that change. The change can be made purely randomly or it can be guided by biochemical knowledge of the interface such as higher shape complementarity of the peptide at the interface, or added hydrogen bond interactions. The assessment step ranges from visual inspections to computational scoring methods to high throughput experimental screening.

**Table 2:** Examples of application of mutagenesis in design of peptide binders.

Starting point	Target	Results
The structure of the APY hairpin bound to the receptor	EphA4 receptor	*K*_d_ as low as 30 nM [47]
C-terminal binding domain of the peptide	C5a Receptor	Series of agonists with a range of ki values as low as 1 *μ*M [48]
Structure of CDK2-cyclin	Cyclin	IC_50_ values as low as 2.1 ± 0.4 *μ*M [39]
Helix segment from HIV-1 gp41 structure	gp41	*K*_i_ values < 0.2 nM after mutating to new a/B AA [49]
pDI inhibitor discovered through phage display [50], binding motif from p53	MDM2/MDMX	*K*_D_ of 0.91 nM for MDM2 and 2.31 nM for MDMX after mutagenesis including ncAAs and crosslinking [51], 4±8 nM and 50±16 nM against MDM2 and MDMX after adding a res that binds in a newly identified pocket [46]
Helical motif of SARS-COV2 bound to Human ACE-2	ACE-2	Showed better computational energies for designed binder [44]
Short helical segment from TRF1_TRFH_-Fbx4_G_ structure	ubiquitin E3 ligase SCF^Fbx4^	kD as low as 23.3 ± 12.8 *μ*M and IC50 of 31.3 *μ*M after mutagenesis [52]

One powerful example of using mutagenesis to improve the binding and selectivity of peptides is the case of helical peptides that bind to the BCL-2 family of proteins. Modifications of this helical motif using a combination of computational mutagenesis, rational mutagenesis, and experimental screening have resulted in a series of potent and selective inhibitors for different members of this family such as Bcl-x_L_ [34], Bfl-1 [35], and Mcl-1 [36]. Mackenzie et. al. showed that statistical information of recurring tertiary structural motifs (TERMs) [37] and their associated energies (dTERMen) can be used to predict the binding energies of the previously designed peptides against BCL-2 family members [38].

To automate the process of incorporating noncanonical amino acids into peptides for improved properties, Andrews et al. developed REPLACE (REplacement with Partial Ligand Alternatives through Computational Enrichment) [39]. This process starts with the structure of the starting peptide in complex with the target of interest. Each amino acid of interest is removed from the peptide and a variety of functional groups are instead docked into the pocket and ranked based on their calculated score function. The best ranking group will be then selected as the new mutation. This process allowed for obtaining new peptides that retained inhibitory activity against CDK2 but with overall less charge, thus a higher chance of being permeable.

In order to identify improved sequences for an existing motif at a binding site starting, Pearce et al. used EvoDesign [40]. In brief, EvoDesign for interface uses evolutionary information from other protein-protein interfaces to design better interfaces. It aligns a library of nonredundant protein interfaces [41] to the interface of interest using iAlign, a computational method developed for aligning and comparing protein-protein interfaces [42]. A multiple sequence alignment (MSA) is then generated from the best scoring aligned interface. Added to this MSA is the evolutionary profile of the scaffold itself that can be used as a guide for designing the peptide. The information from these MSA profiles is used as an energy term that is added to their main energy function, EvoEF, which is then used for optimizing sequences for best binding. The authors recently used EvoDesign in conjunction with an improved energy function, EvoEF2 [43], to design a peptide that blocks SARS-CoV-2 spike protein interaction with human ACE-2 receptor [44].

Rooklin et al. showed that focusing the mutagenesis studies to the unoccupied pockets at the interface between motif and target can result in better binding affinities. To assist with the choice of residues for mutagenesis, they developed AlphaSpace [45], a fragment-centric algorithm for identification and analysis of binding pockets at protein interfaces. At the core of the AlphaSpace algorithm are AlphaSpheres. These spheres are calculated after tessellation of the protein structure. Filtering, clustering, and ranking AlphaSpheres ultimately results in selection of protein pockets. If a pocket is part of a protein-protein interface, the shape complementarity of the residue at the pocket and the pocket can be calculated. If the difference between the volume of the residue and the pocket is large enough to accommodate at least one methyl group, it will be reported and the residue can be mutated to better fit the pocket. In a more recent study, the authors used AlphaSpace to identify secondary binding pockets on protein surfaces near the primary binding sites of MDM2 and MDMX. After identification, they performed a local docking to find out which side chains can best fit that pocket. The original peptide binder was extended to include the newly identified side-chain. The extended peptide had improved binding affinity over the original design (around one order of magnitude). Constraining the original helix and optimizing the linker resulted in final peptides with binding affinities of 4 ± 8 and 50 ± 16 nM against MDM2 and MDMX, respectively (original affinities were around 1 *μ*M) [46].

### 2.2. Stabilizing Motifs Using Crosslinkers

Binding affinity is determined by two factors: the energy of binding at the interface (enthalpic contribution) and the entropic cost of binding as a result of moving from a free state to a bound conformation. The entropic cost is especially prominent for cases where the peptide can adopt multiple conformations in the free form. To reduce this entropic cost and thus enhance binding, many researchers have used crosslinkers that hold peptides in the desired binding competent conformation (Table 3) [53].

**Table 3:** Examples of the use of crosslinking strategy in designing peptide binders.

Starting point	Target	Results
Linear peptide known to bind in the pocket	TLE1	*K*_d_ as low as 3.5±1.9 nM [75]
Originally designed antagonist	C5a receptor	IC_50_ as low as 0.2 nM (cyclization combined with mutagenesis) [76]
Mutagenesis study of native protein-protein interactions	CK2 subunit interaction	IC_50_ as low as 3.0 nM [77]
Antibody loops	Influenza hemagglutinin	*K*_d_ as low as 17±4 nM and IC_50_ as low as 30 nM (after mutagenesis) [74]
Original helical motifs	NHR2-binding (N2B) motif of E-proteins	*K*_d_ as low as 53 ± 20 *μ*M [78]
Structure of B-hairpin at the binding site	CdiI	*K*_d_ of 13 ± 2 *μ*M [79]
Structure of bovine immunodeficiency virus Tat with TAR RNA	HIV TAR	*K*_d_ as low as 1 nM (disulfide cyclization and mutations) [80]
Structure of LXXLL motif (NR box)	Estrogen receptor	*K*_D_ as low as 0.075 *μ*M for ER α and 0.155 *μ*M for ER β [81]
Crystal structure of 14-3-3*ζ* in complex with ESp	14-3-3	*K*_d_ as low as 0.25 ± 0.01 after stapling [82]
Structure of similar CYFIP1 to homologus WASF1	CYFIP1	Showed inhibition in cells [83]
EED binding domain of EZH2	EED	*K*_d_ as low as 264 nM after stapling [84]
Helical domain from Ras interaction with sos	Ras	nucleotide-free Ras with a *K*_D_ of 28±4.8 *μ*M and GDP-bound Ras with a *K*_D_ of 158±16 *μ*M [85]
Previously known binder, StRIP3 [86]	Rab8a	*K*_d_ as low as 7.8 *μ*M after double stapling and mutagenesis [87]
Helical domain of HIF1a interacting with p300	p300	$K_{\text{i}}=3.5\pm 1.2$ *μ*M [88], *k*_d_ as low as 420±35 nM [89]
Loop identified by LoopFinder	Stonin2 and Eps15 interactions	*K*_d_ as low as 0.33±0.01 *μ*M and IC_50_ as low as 2.2±0.3 *μ*M by crosslinking the identified hot loop with *K*_d_ of 18.2±3.4 *μ*M and $\text{I}{\text{C}}_{50}>100$ *μ*M [68]
LC3 interacting region	LC3B	*K*_d_ of 0.12±0.004 *μ*M after stapling and mutational optimization [69]
10mer hot segment identified at the interface	TLR4	Synergistic activation of TLR signaling was observed for cyclized peptides [71]
Short helical segment from TRF1_TRFH_-Fbx4_G_ structure	Ubiquitin E3 ligase SCF^Fbx4^	*k*_D_ as low as 23.3±12.8 *μ*M and IC_50_ of 31.3 *μ*M after mutagenesis [52]
BH3 domain mimics	BCL-2, Bcl-x_L_	Dissociation constants as low as 21±1 nM [90], *K*_d_ of 38.8 nM [91], IC_50_ as low as 5.5±0.8 nM with stapling and β-amino acid [92]
BCL9 helix	β-Catenin	*K*_i_ as low as 0.13±0.05 *μ*M [93]
Helix segment from HIV-1 gp41 structure	gp41	*K*_d_ as low as 7.50±1.70 nM after stapling [94], half life enhanced by an order of magnitude after double stapling [95], IC_50_ as low as 10 nM and *K*_d_ of 17 nM after stapling peptides containing the core binding residues [96]
Structure of p53 helical domain interacting with MDM2/MDMX	MDM2/MDMX	IC50 as low as 2.2±1.1 nM for MDM2 and 3.1±1.2 nM for MDMX [97], MDM2 *k*_i_ as low as 3.21±0.38 nM [98], IC_50_ as low as 6.2±1.5 nM for MDM2 and as low as 210±23 nM for MDMX using a photoinduced cycloaddition [99], *K*_d_ of 160±80 nM for MDM2 [100]

One of the most successful examples of protein crosslinkers are those that keep peptide fragments in a helical conformation, or *staples*. This helix stapling method has successfully been used over the years to generate peptide binders for many targets including MDMX and BCL-2 family of proteins [54–56]. There are now well-defined guidelines regarding which staplers to use and what positions to place them in a helix for better outcome [57, 58].

Similar staples do not exist for β-sheet-containing motifs, but it has been shown that disulfide bonds can be used to stabilize these structures [59]. In addition to inter-strand staples, a series of turn mimetics have been developed to stabilize and assist β-sheet nucleations. D-Pro…L-Pro turn is among the most commonly used motifs for β-sheet nucleation [60]. β-hairpin mimetics using this turn have been widely used as inhibitors of a wide variety of targets such as Viral RNA TAR element [61] and HIV-1 Rev-RRE [62], CXCR4 [63], and for general antimicrobial activity [64]. Aib…D-AA [65] or Aib…achiral AA [66] have also been used as type I’ turn mimetics and are shown to assist with beta sheet nucleation.

Not all protein-protein interactions occur through secondary structure motifs; many are mediated through the so-called *hot loops*, loops at the interface that majorly contribute to binding. Multiple groups are working on using developed or novel crosslinkers to stabilize these hot loops to obtain higher affinity binders for targets of interest. Gavenonis et al. used LoopFinder [67] to identify these hot loops at the protein-protein interfaces [68]. After identifying the loops, the computational ∆G contribution of each residue at the interface was calculated by computational Ala scanning using Rosetta software. Residues with >1 REU (Rosetta Energy Unit) were identified as hot spots. Hot loops were defined as regions with at least 3 hot spot residues, two of which must be consecutive, and average REU per residue of 1 or more. They further developed a binder with 0.33±0.01 *μ*M affinity against stonin2 and Eps15 interactions from a loop discovered in this analysis. In another study, inspired by the natural binder to LC3B, Cerulli et al. used unnatural amino acids and diversity-oriented stapling to obtain high affinity inhibitors of this protein [69].

In a similar approach, London et al. developed a protocol that searches protein-protein interactions and identifies *hot segments* by comparing the calculated binding energy (using Rosetta software) of the fragments to the binding energy of the entire protein to the target [70]. The peptides derived from these protocol were shown to be able to inhibit a number of protein targets including TLR4 [71], ubiquitin E3 ligase SCF^Fbx4^ [52], and gankyrin-ATPase complex [72]. This protocol is available through the Peptiderive server [73] where starting from a protein-protein complex, a hot segment is suggested and if possible, is cyclized via addition of a disulfide bond or through N-to-C cyclization.

One of the most commonly occurring natural loop motifs are those involved in antibody-antigen interactions. These motifs can also be stabilized using crosslinkers to generate peptide binders. Kadam et al. showed that using a series of noncanonical amino acids, they could stabilize and close the binding loop of BnA antibody that binds at the stem region of influenza hemagglutinin, resulting in binders with affinities as low as 17 nM [74].

### 2.3. Grafting Motifs into New Scaffolds

An alternative to using crosslinkers for stabilizing naturally occurring motifs is to use peptide and protein-based scaffolds. In this method the entire structure of the motif or its functional groups is grafted into an already existing natural or designed scaffold (Table 4) [22, 53, 101]. These scaffolds are often highly stable and robust to mutations or insertions of new elements.

**Table 4:** Examples of different structural motifs grafted onto existing scaffolds.

Starting point	Target	Scaffolds	Results
Structure of Rev binding to HIV RNAl helical	RRE RNA	β-Sheet	Mimicked by a β-sheet. *K*_d_ as low as 0.1 *μ*M [62]
RGD motif	Integrin	Knottin	Cell binding affinities as low as 13±3 nM after grafting and affinity maturation [107]
Peptidic antagonist DALK	Bradykinin B_1_ receptor	Cyclotide	Functional stable peptides were obtained [110]
RRKRRR epitope that inhibits interaction of VEGF with KDR receptor	VEGF-A	Katalase B1 cyclotide	Maintained biological activity with enhanced serum stability [111]
A number of known melanocortin 4 receptor activating sequences	Melanocortin 4 receptor	Katalase B1 cyclotide	*K*i as low as 29 nM after grafting [112]
CVX15 peptide	chemokine receptor 4, CXCR4	MCoTI-I cyclotide	EC_50_ as low as 19±3 nM after trying multiple grafting strategies [113]
TCF4 strand bound to β-catenin	TCF4/ β-catenin interaction	β-Hairpin mimic	best $K_{\text{i}}=4.0\pm 0.5$ *μ*M [116]
Structure of helical motif bound at the interface	botulinum neurotoxin B	Miniprotein of size ~45 residues	*K*_d_ as low as 0.5 nM [115]
Structure of helical motif bound at the interface	Influenza haemagglutinin	Miniprotein of size ~45 residues	*K*_d_ as low as 5.0 nM [115]
Structure of SARS-CoV2 spike protein with human ACE-2	Human ACE-2	Miniprotein of size 40-65 residues	*K*_d_ of ~1 nM after affinity maturation [13]

One of the most successful classes of such scaffolds are disulfide-rich peptides [102, 103] such as knottins [104] and cyclotides [105, 106]. These highly rigid scaffolds allow for insertion of loops without losing their structure. Grafting different loops containing the integrin binding RGD motif into knottin scaffolds (*Ecballium Elaterium* trypsin inhibitor) followed by directed evolution resulted in integrin binders with low nM affinities [107]. Since then, it has been shown that engineered Knottins can be used as imaging, therapeutics, or delivery molecules [104, 108]. Engineering cyclotides by grafting different receptor binding loops into them resulted in peptide binders for a number of GPCR receptors [109] including bradykinin receptor [110], VEGF-A [111], melanocortin 4 receptor [112], and chemokine receptor 4 [113, 114].

In addition to interactions involving loops, secondary structures can also be grafted onto other scaffolds. Bigger peptides with folded structures called miniproteins have recently been used for stabilizing these structured motifs known to interact with protein targets of interset. This method has been successfully used to generate binders with very high affinity and selectivity against many targets including Influenza HA [115], and Botulinum neurotoxin B [115].

The secondary structure motifs have also been incorporated into smaller peptides. For example, Blosser et al. used β-hairpin mimetics to stabilize an extended β-strand that mediates TCF4/ β-catenin interaction [116]. They started their work by first identifying the minimal interacting sequence that could compete with the native TCF4 motif (*K*_i_ of this fragment was 57±18 *μ*M). A rationally designed strand was then added to this strand with stabilizing hairpins in between. This new strand was designed to stabilize the structure, enhance solubility, and generate additional interactions. This process led to the generation of binders with 10-fold improvement in affinity (best $K_{\text{i}}=4.0\pm 0.5$ *μ*M).

## 3. De Novo Design of Functional Peptides

**Figure 4:**
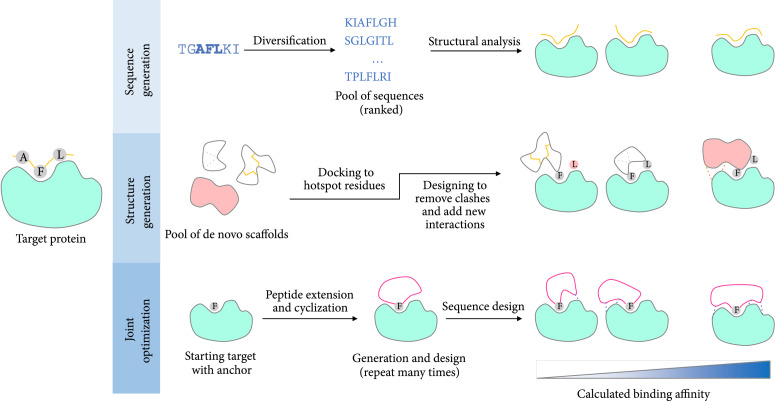
Schematic overview of de novo design methods to generate peptide binders. De novo design of binders can be broadly categorized into three methods: (a) de novo generation of sequence: In this method, first a diverse pool of sequences is generated and then the best binders are selected based on their sequence ranking followed by binding energy calculation based on downstream structural analysis. (b) De novo generation of structure: In this method structured scaffolds are first generated. They are then used to present and stabilize a select set of hotspot residues at the interface. (c) Joint optimization of sequence and structure: In this method the sequence and structure are generated in one step. This method often starts with a functional group at the interface called the anchor. The peptide structure is generated in the context of the protein pocket, the sequences are optimized, and the structure is adjusted based on the sequence to obtain the best binders. Repeating this process multiple times results in a pool of potential binders.

As its name suggests, *de novo design* of peptides refers to the process of designing peptides from scratch. The term de novo has been used for a number of different approaches. In this review, we will use de novo design for cases where the generated peptide does not stabilize a known naturally occurring motif at the interface. The de novo design methods to generate functional peptides can be divided into three categories (Figure 4): (a) methods that generate sequences followed by conformational modeling/sampling, (b) methods that generate structures first and make sequence modifications later, and (c) methods that sample sequence and structure simultaneously.

### 3.1. De Novo Generation of Sequences

**Figure 5:**
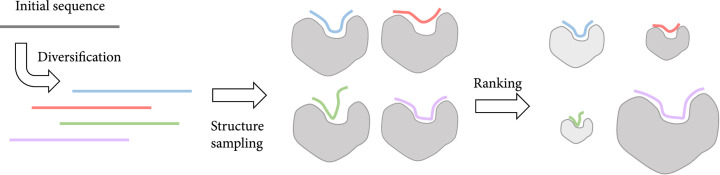
General overview of de novo design methods that sample the sequence space. The design process starts with a diversification step where a pool of sequences is generated. These sequences then go through a structural modeling step where they can be ranked. The different sizes in the right panel are proportional to the rank (the best model is the biggest). The best ranking sequences are then tested or fed as the initial sequence for another round of design.

The sequence-based methods (Figure 5) often start from a known binder sequence or a pool of known binder sequences and generate diversity in this pool to obtain the best binder. Due to the large space of potential sequences, often some constraints are applied to the generated sequences, and/or some optimization methods are used. The type of applied constraints used or optimization methods are often determined by the information at hand and the target of interest. The generated sequences are then ranked to find the best candidates. This ranking can be done in a number of different ways, but often requires considering the structure of the peptide-target complex or its model. While case-specific, methods for de novo sampling of sequences can be generalized to other similar cases or inspire the development of new general methods. Thus, in the following section, we describe examples of these methods for de novo design of peptides.

To design peptides that can selectively inhibit EZH2, Smadbeck et al. have developed a multi-stage framework [117]. In the first stage, a series of potential peptide sequences were generated starting from the native peptide binder to EZH2. The generated sequences were then ranked using an energy minimization method that ensured stability. This was followed by assessing the ability of the designed peptides to match the structure of the template peptide, determined by measuring the fold specificity of each sequence for the target structure. The top sequences were then subject to binding affinity calculation using a method previously reported by Lilien et al. [118]. The binding affinity for the final 45 candidates were then calculated by RosettaDock and the top designs were validated experimentally. Most of the predicted peptides were able to inhibit EZH2 with affinities in the low *μ*M range. One of the designed peptides, SQ037, showed an IC_50_ of 13.57 *μ*M, higher than that of the existing small molecule inhibitors of EZH2, which are in the nanomolar range.

Unal et al. [119] also addressed the de novo design of binders in a multi-step approach. In the first step, the binding site on the protein of interest was determined using a coarse-grained Gaussian Network Model [120–122]. This step was followed by an arbitrary docking of a peptide (a known interacting partner or poly-Ala if the information on the partner didn’t exist) on this binding surface using Autodock. The next step involved calculating the binding energies of all possible amino acid pairs at the binding interface. The transition probabilities (probability of a particular amino acid occupying a position, given another amino acid is in the partner position) in the Markov based model was calculated and was used to identify the best peptide binder. They validated their method by testing binders against five different proteins, including HIV-1 protease and scytalidocarboxyl peptidase B protein. The experimental data showed improved binding affinity compared to the naturally occurring peptides. For instance, the tripeptide Trp-Tyr-Val, designed by their algorithm showed a binding affinity of −9.59 kcal/mol against HIV-1 protease. In reference, the binding affinity of a known inhibitor to HIV-1 protease, Glu-Asp-Leu, determined by the same method is -7.66 kcal/mol.

**Figure 6:**
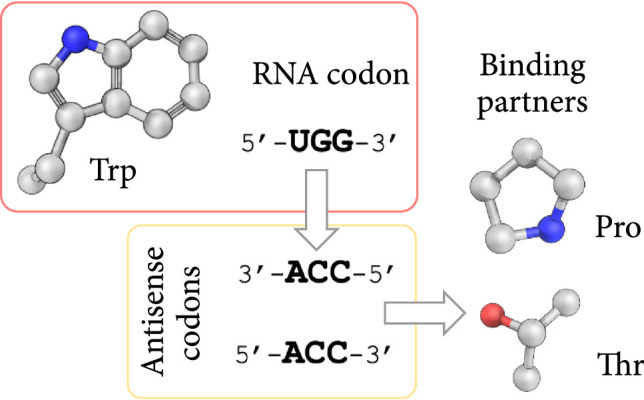
Schematic representation of sense-antisense amino acid pairing theory. The overall hypothesis behind this theory is that when choosing amino acids to bind to a target surface, amino acids that are antisense of those at the interface tend to bind with higher affinity than randomly selected amino acids. Whether two amino acids are sense-antisense is determined from their tRNA codons as shown in the figure.

Li et al. [123] developed a method for the design of peptides that can inhibit VEGFR-3. First, they identified the binding pocket on the outer surface of the VEGFR-3 protein using FT Map [124]. They then constructed a library of 576 peptide sequences based on Mekler–Idlis sense-antisense amino acid pairing theory (Figure 6) [125]. In the next step, molecular docking simulations and conformational analyses were performed to rank these peptides with respect to their binding interactions with VEGFR-3. The best ranking peptides were then synthesized and further experimentally evaluated. Cell binding experiments identified several peptides, including CP-7, that could selectively target VEGFR-3. For example, designed peptide CP-7 (CVKTFDP) could bind to 94.6% of the A549 cells in the experimental setting compared to 57.8% bound by their positive control (LARGR).

### 3.2. De Novo Generation of Structure

In this method, researchers first generate peptides with predefined structures. These peptides will then be used as scaffolds to bind to a target surface of interest (Table 5). To guide the binding and increase the probability of success, these scaffolds often contain hotspot residues known to bind to the target of interest. The design process needs to ensure that these hotspot residues are represented in an orientation that will be competent for binding. Thus, these approaches are more hands-on than those described in sections 2.2 and 2.3 where an entire secondary structure or loop is crosslinked or grafted.

**Table 5:** Examples of de novo designed scaffolds used for binding to protein targets.

Designed scaffold	Target	Results
Foldamers	MDM2	IC_50_ as low as 24 nM [127]
Foldamers	Vitamin D receptor	*K*_d_ as low as 0.12 *μ*M [127]
Terphenyl scaffolds	Bcl-X_L_	*K*_i_ as low as 0.114 *μ*M [128]
De novo coiled coils	MCL-1	IC_50_ as low as 0.09±0.005 *μ*M [130]
β [3]-peptide	gp41	EC_50_ as low as 5.3±0.5 [134], one variant was later improved by near 7 folds through mutagenesis [135]
β-peptide	hDM2	IC50 of 80.8 ± 3.2 nM [136], further improved to as low as 27.6 nM for MDM2 upon mutagenesis [137]
β-hairpin	HDM2	IC_50_ values as low as 0.14±0.06 after optimization [129]
Designed peptide-peptoid	TCF/β-catenin	Best binder had IC_50_ of 5.44 ± 0.82 *μ*M [133]
Miniproteins of ~65 residue	SARS-COV2	5 pM after affinity maturation [13]

It has been shown that in cases where the interactions between the motif and the target are mainly through side-chains, presenting these side-chains in the right orientation is enough to achieve binding. These cases often involve secondary structure features such as α-helicx and β-sheets. A library of peptide-like *foldamers* developed that can fold into stable alpha helices and β-sheet mimetic purely based on their backbone propensities, thus allowing open sites for presenting functional side-chains in the orientation similar to the motif.

**Figure 7:**
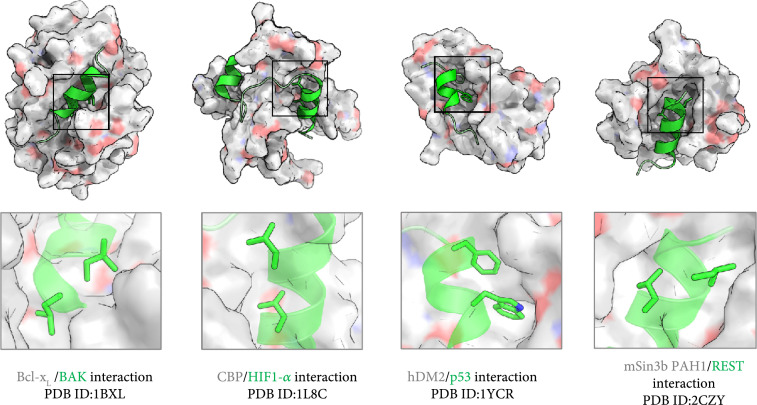
Helical motifs are prevalent at protein-protein interfaces. Examples of helix-mediating protein-protein interactions are shown. The sticks show hot spot residues that make a major contribution to binding. The boxes at the bottom row are zoomed views of the hot-spot residues at the binding interface (boxed regions in the overall figures shown in the top row). The helices are shown as transparent cartoons in the bottom panel for clarity.

The abundance of helical motifs at protein-protein interfaces (Figure 7) [126] created a large space for the use of α-helix mimicking foldamers with many successful examples reported to date. One successful example of α-helix mimicry are peptide-oligourea hybrids, used to inhibit a variety of proteins such as MDM2 and vitamin D receptors [127]. Another example is the use of Terphenyl scaffolds as helix mimetics to generate binders against Bcl-x_L_ and Bak interactions [128].

Small β-hairpins can also be used to mimic the placement of key residues presented by helical motifs. For example, Fasan et al. used a small β-hairpin to stabilize two hot spot residues at the interface of p53 with HDM2. After a series of mutational studies, they obtained a final peptide with binding affinity in the order of 100 nM [129].

De novo designed alpha-helical coiled-coil structures are another class of scaffolds that can be used to stabilize interactions at the protein-protein interfaces. Fletcher et al. [130] have designed de novo parallel homodimeric and heterodimeric α-helical coiled-coil scaffolds to target and disrupt the interface of the MCL-1/NOXA-B by stabilizing key interacting residues. The critical residues in NOXA-B that are involved for binding were first identified by computational alanine scanning, using Robetta [131], and classified into a short motif and an extended motif. These residues were then grafted on the sequence of the de novo peptide CC-Di [132] to get a series of possible hybrid designs. These designed peptides were synthesized in the lab and were evaluated to check for their purity and stability. The inhibitory capabilities of these hybrids were checked by fluorescence spectroscopy and it was concluded that their binding to MCL-1 stabilized these coiled-coiled assemblies.

Hotspot residues at the interface that guide the design of peptide binders can be generated computationally. For example, Cao et al. used miniproteins as their starting scaffolds to present computationally generated hotspot residues and developed inhibitors with IC_50_ as low as 5 pM against SARS-CoV-2 spike protein [13].

The de novo design of binders can also start in a hotspot-free manner. In this method, researchers start with searching a library of existing peptides for shape-complementarity to the surface of interest, and enhance binding through sequence design. For example, starting with the structure of TCF/β-catenin interaction, Schneider et al. searched for the best scaffold from a series of previously designed high-resolution peptoid or peptoid-peptide hybrid scaffolds that can best mimic and inhibit the β-hairpin motif at this interface [133]. After finding the best scaffold, they performed a series of computational mutagenesis and tested the best ranking binders experimentally. Their best binder had an IC_50_ of 5.44±0.82 *μ*M and showed inhibition in cells as well.

### 3.3. Simultaneous Optimization of Sequence and Structure

Simultaneous optimization of sequence and structure, while intuitive, can be computationally expensive. Molecular dynamics (MD) simulation methods that take into account structural changes are often time-intensive, and including mutations in the process is not feasible. De novo methods to design sequences that stabilize a given structure can include slight backbone movements, but large-scale movements that can be observed in MD are often not possible as the sequence optimization problem for a single rigid backbone is in itself an expensive problem. However, for very small peptides, these two processes can be combined to design peptides with pre-defined structures or function.

While linear peptides are often too flexible to be accurately designed, constrained peptides such as cyclic peptides have fewer degrees of freedom and can be designed computationally to adopt predefined shapes. In 2018, Slough et al. designed rigidly structured peptides (pentamers) in an aquatic environment using MD [138]. They simulated a library of cyclic pentapeptides differing only in two residues (excluding the Pro) using BE-META MD. They used the result of these simulations to develop a score function for predicting structures of cyclic pentapeptides. This score function was developed based on the preference of 2-mer pairs to adopt a given conformation during the simulation. One of the peptides in the library was well-structured, and its structure was confirmed by NMR as well. However, most other sequences in the library were not well-structured. They observed that some turn combinations that build up these pentamers score poorly; thus, it is very unlikely to obtain a structured peptide containing these turn combinations using the substitutions they considered initially. Therefore, the authors proposed using ncAAs to stabilize these and other conformations. They also suggested that their method can be extended to larger peptides and other types of cyclic bonds.

**Figure 8:**
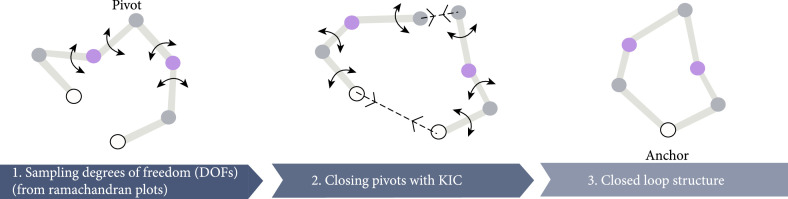
A schematic overview of sampling cyclic peptides with generalized kinematic loop closure. In the first step, the torsional degrees of freedom of all but three residues (pivots) are randomly sampled. In the second step, the torsion angles of pivots are calculated to find a closed solution. The anchor position will remain unchanged.

In addition to MD-based methods, Rosetta software has been used to design structured cyclic peptides. The Rosetta-based design takes advantage of a method called generalized kinematic loop closure (GenKIC, Figure 8), which is inspired by a robotic approach used to calculate accessible conformations for a constrained object (e.g., a finger fixed at joints) [139]. Using this method, the authors sampled a large set of potential cyclic backbones starting from poly-Gly sequences. After clustering these sampled conformations, they performed sidechain assignment by restricting D- and L- amino acids to positions with phi > 0 and phi < 0, respectively. By preventing the use of Gly in the design due to its flexibility, the authors made sure that the assigned sidechains were optimized for stabilizing their backbone conformation. NMR studies confirmed that these peptides indeed take the conformations they are designed for, showing a success rate of >70%. An analysis of the computationally generated library of 7-10 residue cyclic peptides identified more than 200 stable structures using this method [140].

Compared to MD-based methods, the Rosetta-based method has the advantage of being very fast; thus, it can cover near-complete space of D- and L-amino acid combinations in peptides of size ranges as large as 11 amino acids [140]. Additionally, it can be used in line with other design modules to sample both conformations and sequences of cyclic peptides at the target protein surface to design peptide binders. To use this method for the design of target-specific cyclic peptides, a known binding molecule is used as the starting point or anchor on the target surface; then, the backbone is expanded and circularized at the pocket. The conformations of cyclic peptides are sampled using GenKIC, and for each conformation, sequences with maximum favorable interactions to the protein surface are designed. After sidechain and rotamer refinement, the computationally generated designs are filtered based on a number of interface metrics such as shape complementarity, and ∆G of binding, and the best designs will be evaluated using downstream experimental approaches [141].

Using this method, several groups have been able to successfully design peptides for therapeutic targets, such as programmed cell death 1 (PD-1) receptor [142], antibiotic resistance factor NDM1 [143], and histone deacetylase (HDAC-2 and HDAC-6) enzymes [144]. Although the overall design process of these studies is the same (described above), there are differences in each. Mulligan et al. introduced energy terms that could guide the design not just by the overall score but also by the interface quality. They also showed that more rigid peptides have better binding, as the flexible peptides have a higher enthalpy and will negatively affect *Δ*G of binding [143]. Hosseinzadeh et al. showed that high-affinity binding could be obtained with nonstructured peptides. They also added an exhaustive search near the binding pocket to find the best interacting residues before extending the peptide and showed it highly increased the success rate [144]. The best designed peptides for PD-1 had a *K_d_* of 30 *μ*M and 102 *μ*M, which could outcompete the natural ligand. Six out of seven designed peptides chosen for experimental validation of NDM1 inhibition had lower IC_50_ values than D-captopril control and the top peptide had an IC_50_ value of 1.2 ± 0.1 *μ*M, 50 times lower than the control. 17 of 39 peptides tested for HDAC-2 had IC_50_ values of 100 nM or lower. The best HDAC-6 and HDAC-2 binders showed IC_50_ values of around 4 and 9 nM, respectively, three orders of magnitude better than the starting anchor.

## 4. Machine Learning for Design of Functional Peptides

While the field of protein structure prediction has been revolutionized by the recent advances in deep learning [145, 146], predicting structure or function of peptides, or designing new functions is still a challenge. In this section, we first review the successful application of learning methods for the prediction of structure or function of peptides (Section 4.1). We will then describe recent advances in designing new peptides using machine learning (ML) in Section 4.2.

**Figure 9:**
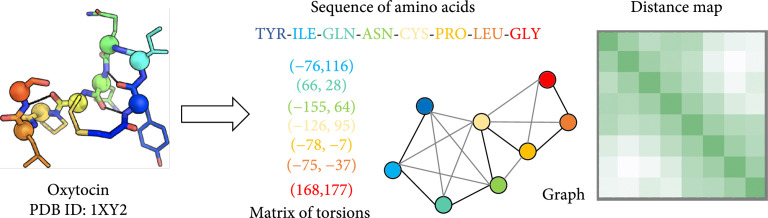
A peptide can be represented in many possible ways. Different representations take into account different features of the peptide. For example, sequence input does not have any explicit information about structure. To each input representation, a different set of learning methods can be applied.

The learning methods that are applied for design and prediction heavily depend on the peptide representation used as well as the type and number of available data in the training set (Figure 9). Thus, our main effort in this section is to cover a wide variety of representations and methods to cover the wide scope of the field.

### 4.1. Predictive Methods

#### 4.1.1. Prediction of Structure

Recent developments in computational algorithms have resulted in great progress towards designing cyclic peptides that stabilize a predefined structure and fold into one conformation (see section 3). However, many native peptides can adopt multiple conformations in solution. The ability to predict these conformations is helpful in the design of peptide fragments with desired functions and in predicting their function. But, predicting the various conformations that cyclic peptides can adopt in solution is still a challenge.

One advancement in this area is StrEAM that predicts peptide conformations through combining MD simulations with deep learning. Miao et al. [147] have tailored a metadynamics method using explicit solvent for modeling cyclic peptide conformations. In this method, they used a bias-exchange metadynamics process to capture the transitional motions of cyclic peptides. While simpler models that do not consider solvent can be used to design cyclic peptides, to accurately model the solvent-exposed backbone C=O and N–H bonds which are sometimes associated with caged water molecules, one needs to use an explicit solvent model. In this algorithm, they have described the structure of penta-peptides by assigning the (phi, psi) angle of each residue to one of the ten different bins in the (phi, psi) space, resulting in a string of torsion bins. They then used results from multiple MD runs on several peptides and interactions between residues i,i+1 or i,i+2 as inputs to a neural network predictor. This algorithm could efficiently predict structural ensembles of cyclic peptides with MD simulation. StrEAMM can also be extended to larger cyclic peptides due to inclusion of the above mentioned longer range interactions.

#### 4.1.2. Prediction of Function

In addition to structure prediction, computational techniques such as probabilistic models, molecular modeling, and deep learning have been extensively used in screening novel biologically active peptides and predicting their functions. In the following sections, we discuss examples of these applications.

**Figure 10:**
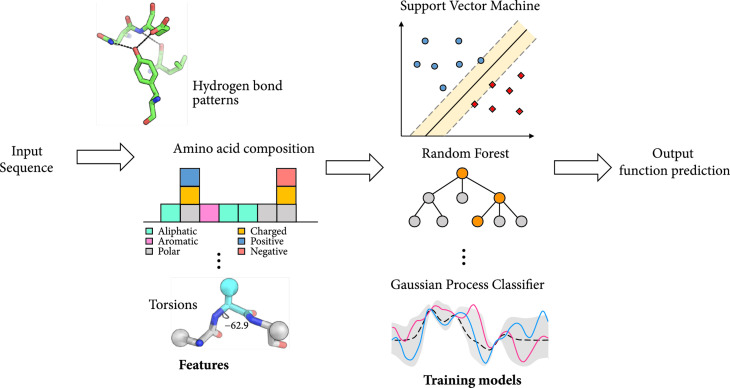
General schematic for prediction of cell penetration potency of given peptidic sequences. The prediction algorithm considers an input sequence of the peptide of interest. Various features like amino acid composition, number of rotatable bonds, hydrogen bonds, torsions, and others are extracted from the sequence to train the prediction model (SVM, random forest, Gaussian process classifier, among others). The model then predicts the cell penetrating potency of the input sequence.

*(1) Prediction of cell penetrating cyclic peptides*. One function of interest for peptides is their cell penetration potency and extensive work has been done in developing predictors for cell penetrating peptides (CPPs). The methods developed for prediction of CPPs (Figure 10) can be adapted for prediction of other peptide functions. A summary of the examples discussed in this section is shown in Table 6.

**Table 6:** An overview of some of the methods developed to predict CPP function. Note that the reported accuracies are not on similar datasets and thus cannot be compared.

Method	Training Set	Features	Result	Accuracy
CellPPD [148]: SVM, MEME/MSAT	708 experimentally validated, unique CPPs from the CPPsite database	Amino acid composition, Dipeptide Composition, Physicochemical Properties of amino acids	Detailed 120 CPP motifs identified from their dataset	97.40%
SkipCPP-Pred [150]: K-skip-n-gram model, RF classifier	1855 experimentally validated CPPs from CPPsite 2.0	Input sequences	High quality dataset CPP924 with reduced redundancy	90.6%
CPPred-FL [152]: RF classifier	Benchmark dataset CPP924 containing 924 peptide sequences	6 features based on class information and 19 features based on probabilistic information	New feature representation algorithm that includes all sequence based feature descriptors	91.2%
BChemRF-CPPred [154]: MLP architecture, SVM, Gaussian process classifier	CPPsite 2.0, C2Pred server	Structure based descriptors including molecular weight, number of rotatable bonds, topological polar surface area, number of hydrogen bond donors and acceptors, etc. Amino acid composition-based descriptors including dipeptide composition. Pseudo-amino acid composition related to hydrophobicity, hydrophilicity, side chain mass, etc	Evaluated the influence of structural and physicochemical properties in the permeability of peptides	89.62%

Gautam et al. [148] have developed a web-server, CellPPD, that can predict and design CPPs. This web-server uses a support vector machine (SVM) for prediction of CPPs. A dataset of 708 CPPs from CPPsite was used as the input along with features like amino acid composition, dipeptide composition, and physicochemical properties. In addition to SVM-based predictors, their web server also uses a hybrid motif-based predictor using MEME/MSAT program [149] which can achieve an accuracy of 97.40% for their dataset, greater than the SVM based or MEME/MSAT program alone. To understand the dominance of certain amino acids in CPPs, they calculated and compared the amino acid composition in the CPPs and concluded that residues like Arg, Lys and Trp were present abundantly in CPPs. The authors also analyzed the amino acid distribution in different positions and concluded that while basic residues like Arg and Lys were present throughout the sequence, the N- and C- terminals preferred different sets of residues. A list of 120 CPP motifs present in their dataset has also been provided. The program can also perform point-mutations on a user-specified sequence to suggest modifications with higher CPP properties.

Wei et al. [150] developed an improved predictor called SkipCPP-Pred. SkipCpp-Pred uses sequences collected for the CPPsite 2.0 as an input and converts them to a feature through application of the k-skip-n-gram model. [151] The k-skip-n-gram model is based on a language processing probability model that tries to predict the context or surrounding words in a string from a target word and thus can capture the correlation between residues. These features are then used to train a random forest classifier that performs the prediction. The accuracy of prediction by SkipCPP-Pred was 90.6% compared to 87% and 83.7% for CellPPD-DC and CellPPD-BP, respectively. In addition to developing this model, the authors also created a high-quality dataset with reduced sequence redundancy to decrease performance bias.

Qiang et al. [152] developed a tool called CPPred-FL. This tool uses random forest classifiers with composition and physicochemical properties of amino acids as descriptors. To address some of the challenges regarding proper feature representation for CPP prediction, the authors employed a new feature representation algorithm (including a learning and an optimization step) that transforms high dimensional feature space into a low dimensional space. With inclusion of their top 19 features, their model can achieve a performance with 92.1% accuracy.

In 2019, Fu et al. [153] developed a machine learning based method to predict CPPs. They considered four features based on amino acid composition, such as frequency of amino acid and composition of amino acid group pairs, and trained their SVM model to classify CPPs. Their predictor showed an accuracy of 92.3% in predicting CPP in the training dataset, better than existing state-of-the-art methods like CellPPD and SkipCPP-Pred.

Further improvement of existing algorithms has led to the design of better classifiers over the years. For instance, de Oliveira et al. [154] have developed an ML-based framework termed BChemRF-CPPred that is capable of differentiating CPPs from non-CPPs. This framework used different classifiers including an artificial neural multilayer perceptron or MLP architecture, SVM and a Gaussian process classifier. It then employed a voting classifier to make the final prediction based on the prediction of each one of these models. In contrast to other methods that use sequence features, this method uses structure-based descriptors that can be related to the permeability of biological membranes such as molecular weight, number of rotatable bonds, topological polar surface area, net charge, and number of negatively charged amino acids. The inclusion of structure-based features improved the accuracy of the model compared to the model based on sequence input only (from 86.5% to 90.66%). They also compared their method with existing methods like CPPred-RF and SkipCPP-Pred using an independent dataset of natural peptides (60 CPPs and 75 non-CPPs). BChemRF-CPPred obtained an accuracy of 89.62% in comparison to CPPred-RF and SkipCPP-Pred’s 68.88% and 62.58% respectively.

*(2) Prediction of anti-cancer peptides*. Peptide-based cancer treatment is one area of interest because of peptides’ high specificity, low toxicity, and membrane permeability. One of the hurdles to predict the biological function of anti-cancer peptides is the lack of sufficient experimental data. To overcome this limitation, Chen et al. [155] have designed a deep learning method termed xDeep-AcPEP that can predict anti-cancer activity of peptides against six different tumor cells: breast, colon, cervix, lung, skin, and prostate. In this method, multi-task learning (MTL) was explored where multiple neural networks were trained independently for individual prediction tasks. For training their model, the authors considered the experimentally verified anticancer peptides (ACP) from the CancerPPD database. Four descriptors AAINDEX, BLOSUM62, ZScale (which reflects the physicochemical properties of amino acids such as lipophilicity, steric properties and electronic properties of amino acids) [156] and one-hot-encoding were used in combination to encode a sequence at the residue level. The encoder model architecture was chosen as a combination of convolutional layers with ReLU activation functions, average pool layers, batch normalization layers and Max Pool. Six convolutional neural network (CNN) models were trained for six tissue-specific ACP dataset. Comparisons between single-task learning (STL) and MTL based models showed that MTL increases the generalizability of the model. The model was tested on 19 recently published ACPs to test its predictive power. The results showed that these models were capable of accurately predicting the anticancer properties of highly active peptides but were less accurate for inactive peptides or peptides with weak activities.

*(3) Prediction of antimicrobial peptides*. Antimicrobial peptides (AMP) are attractive potential substitutes for antibiotics to fight against antibiotic resistant infections. Extensive study on AMPs have led to considerable efforts to develop computational methods for accurate prediction of AMPs from conventional machine learning models to deep learning (see Table 7).

**Table 7:** Overview of methods developed for prediction of AMP activity. Note that the reported accuracies are against different benchmarks and thus cannot be compared.

Method	Training set	Result/Output	Accuracy
14 ML algorithms including RF, CART, SVM, K-nearest neighbor, Logistic Regression (LOGREG), Adaptive Boosting (AB) [157]	HemoPI-1, HemoPI-2 and HemoPI-3	Predicts hemolytic nature with more accuracy than hemolytic activity	HemoPI-1: 94-95% HemoP2/3- 75-77%
AI4AMP [158]: PC6 encoded Deep Learning Model	APD3, LAMP, CAMP3, DRAMP	Considers physicochemical properties of aminoacids as features	90%
Deep-AmPEP30 [159]: CNN, Reduced AAC (RAAC)	Subset of a training set used in AmPEP [160] (experimentally validated AMPs from APD3, LAMP, CAMPR3)	Predicts AMP activity of short peptides from genomic DNA.	77.13%
AntiBP [161]: ANN, Quantitative Matrices (QM), SVM	486 peptides shorter than 61 residues from the APD database	Preference for certain residues at the N- and C-terminals that demarcate them from non antibacterial peptides	ANN based: 88.17%, QM based: 90.37% SVM based: 92.11%
AMPA [162]: Web application to assess the antimicrobial domain in protein. Can serve as template for AMP design	High throughput screening results from amino acid substitutions on bactenecin 2A	Quick discovery of new AMP patterns in protein sequences	accuracy of 85% and a sensitivity of 90% [163]
AVPpred [164]: SVM	1245 experimentally validated antiviral peptides	Predicts highly effective AVPs	86%
iAMP-2L [165]: Pseudo-amino acid composition, Fuzzy K-Nearest neighbor	Curated set of AMP and non-AMP peptides collected from Uniprot	Predicts if a given sequence is an AMP or not and if yes, the next classification is done on the type of AMP	92.23%
AMP_Scanner [166]: Deep Neural Network with the Keras framework (http://www.keras.io) using a sequential model and a TensorFlow deep learning library back-end	APD vr.3 database	This model automatically extracts expert-free features and hence removes the reliance on domain experts for feature construction	91.01%

One of the main problems with AMPs is their hemolytic nature. Hemolysis disrupts the erythrocyte membrane and decreases the lifespan of RBCs. Thus, it is very important to identify and predict the hemolytic activities of AMPs. Plisson et al. [157] have developed ML methods to discover AMPs with low hemolytic properties. The model was trained on three publicly available datasets (HemoPI-1, HemoPI-2, and HemoPI-3) and employed 14 ML algorithms, including CART, random forest, and SVM, for binary classification. The model was tested on 3081 natural antimicrobial peptides from the APD database and 317 known hemolytic antimicrobial peptides were considered for validation of the model. 56 physicochemical properties, including aromaticity, hydrophobic ratio, net charge, and bulkiness were used as features for the model. The algorithm predicted the hemolytic nature of the peptides with greater accuracy compared to the extent of the hemolytic activities.

Lin et al. [158] developed a web-based server called AI4AMP that can accurately predict the antimicrobial potential of a given sequence. In this work, they proposed a new protein encoding method called PC6 (physicochemical component 6) that included features like hydrophobicity, volume of side chains, and isoelectric point. The server has an precision of 90%.

Yan et al. [159] developed a deep learning based tool called Deep-AmPEP30 for prediction of AMP activity of short length peptides from genomic DNA. The prediction is based on a reduced dataset and uses a deep CNN. They validated their model by identifying a 20 amino acid long peptide from the genome of the fungus, *Candida glabrata*, and the activity of this peptide was verified through experiments.

*(4) Prediction of peptide-protein interaction*. Many peptides exert their functions through binding to a protein partner; therefore, predicting a peptide-protein interaction is of huge interest. Lei et al. [167] have designed a deep learning framework, called CAMP, that can predict whether a peptide and a protein pair with each other. CAMP uses peptide-protein complexes from the PDB and the known peptide-protein pairs from the DrugBank Database. It then uses PLIP [168] to extract peptide-protein pairs that interact via noncovalent interactions. The model then creates feature profiles for both the peptide and the protein partner using structure and physicochemical properties of residues. It also uses PepBDB [169] to extract labels for residues that are involved in binding. The features for the peptides and proteins are then fed into a CNN layer and a self-attention module to predict the interacting pairs as well as peptide residues that are involved in making the interaction. Their model had a mean of AUC value of 0.803 and mean of average MCC value of 0.489. They showed that their model can correctly predict known protein-peptide pairs such as GLP-1 receptor and Semaglutide.

### 4.2. Generative Methods

One of the key limitations in application of learning-based methods for the design of functional peptides has been the lack of data for training. To overcome this limitation, researchers have developed creative approaches to link data generation through (semi-)high throughput methods with learning to improve the process of design. It has been shown that learning from a representative subset, even if small compared to the entire space, can result in significant improvement in the success rate of the original methods [170]. In this section we will first discuss examples of these methods (Section 4.2.1) and then move on to fully computational methods that tackle design using available databases (Section 4.2.2). The inputs and learning methods highly depend on the function of interest and available data. Thus, our focus in this section is to review cases that cover a variety of inputs and models. A summary of the examples discussed in this section is shown in Table 8.

**Table 8:** A general overview of learning-based methods that have been applied to the task of designing functional peptides.

Task	Input data	Model	Output
Binders for Bcl-x_L_, Mcl-1, or Bfl-1	Set of 10,000 previously generated binders	SORTCERY [171]: Support vector machine for prediction, applied to a larger sequence library	36 high affinity binders for each target with *K*_i_ as low as 5.7±1.2 nM for Mcl-1, 1.32±0.18 nM for Bcl-x_L_, and 6.6±1.0 nM for Bfl-1
Finding peptide hits for Sfp and AcpS	Experimental data of known hits and fragments that don’t bind, each round new data was added	POOL [172]: Naive Bayesian	Selective binders for Sfp or AcpS after 4 rounds
Design of cell permeable peptides	600 PMO-miniprotein conjugates	RNN for generation and CNN for prediction [173]	Synthesized and characterized 12. Highly active for macromolecule delivery
Peptides with anticancer properties	Curated peptides from CancerPPD that target breast or lung cancer, pepCAST descriptors were used to find features	CPANN [174]: counter propagation artificial neural network for prediction and modLAMP for generation	6/15 peptides predicted to have anticancer activity indeed showed anticancer activity
Design of chitin binding peptides	21 million amino acid sequences taken from the Pfam database and 20000 sequences from the SCOPe database	Two Bi-LSTM each with 1024 hidden layers [175]	Interactive residues in the two predicted peptides matched experimentally known ones
Designing nonhemolytic AMPs	DBAASP database	RNN for classifying active, inactive, hemolytic, and nonhemolytic sequences [176]	12/28 were active

#### 4.2.1. Design-Test-Learn Methods

Jenson et al. [171] used a combination approach to generate functional peptides with high affinity and selectivity to three different targets: Bcl-x_L_, Mcl-1, or Bfl-1. They used a high throughput method called amped SORTCERY to measure the binding affinity of a library of around 10,000 peptides selected from previously designed combinatorial libraries against their target proteins. They then employed support vector regression to fit this experimental dataset. Next, they applied this predictor to their entire library of nearly 27,600,000 peptides as well as a large input dataset of 10^14^ potential peptide sequences. To search new sequences with desired binding features (for example high affinity to one while selective against other members), the authors applied an integer linear programming solver. They were able to generate 36 peptides that could bind with high affinity and specificity to either of the three proteins, namely, Bcl-x_L_, Mcl-1, or Bfl-1. The advantage of such a sequence-based model is the faster process of searching the sequence space to design the structure of the peptide.

Tallorin et al. [172] have developed a hybrid computational and biochemical method that can rapidly optimize peptides for a given biochemical function. This method, named POOL (Peptide Optimization with Optimal Learning) iteratively considers experimental data and fits them into a mathematical model to predict new hit sequences. In its core, POOL is a Naive Bayes algorithm that learns the probability of a given sequence to be a hit based on the training data. The initial database given to POOL was a combination of known peptide and protein fragments that were substrates for protein targets of interests Sfp (surfactin phospho-pantetheinyl transferase from *Bacillus subtilis*) and AcpS (holo-acyl carrier protein synthase from *Streptomyces coelicolor*) as well as a set that were known to be inactive. After each experimental round, the new data was added to this dataset. At each round, POOL is asked to predict a certain number of potential peptide hits such that the total probability of obtaining at least one hit is maximized. One key component of POOL is the fact that after it identifies each hit, the process of finding the next hit assumes the previously recommended peptides are not hits. This assumption ensures that multiple models are considered for predicting new hits. Compared to traditional predict-then-optimize methods, POOL can more effectively sample the space of functional peptides due to a better balance between exploitation and exploration, as suggested by the authors. After four rounds of learning and testing, the authors obtained short peptides that could selectively label AcpS or Sfp.

Schissel et al. [173] have combined high throughput experiments and deep learning approach to design de-novo abiotic nuclear targeting miniproteins. Their model was trained on a dataset consisting of unnatural residues and structures. The authors synthesized and tested a library of 600 phosphorodiamidate morpholino oligomer (PMO)-miniprotein conjugates using a combination of abiotic peptide molecules. These sequences were labeled with their experimental activities and were used to train their CNN-based predictor. Their generator was a nested LSTM neural network architecture. Sequences generated using this generator were then fed into a cycle of prediction and optimization (using a genetic algorithm) to reach new sequences with high likelihood of delivery across cell membranes. The model predicted hundreds of miniproteins and they synthesized and characterized 12 of them. The predicted activities for nearly all the sequences matched the experimental values.

#### 4.2.2. Fully Generative Models

De novo design of anticancer peptides (ACP) holds importance in cancer therapeutics. In this context, Grisoni et al. [174] have presented a counter propagation artificial neural network (CPANN) [177] to generate peptides that have potent anticancer properties. The counterpropagataion network [178] is a statistically optimal self-programming lookup table. Databases of peptides targeting breast and lung cancer were curated manually from the CancerPPD database and used to train the CPANN model. The pharmacophore features of the peptides were determined using the pepCATS descriptors. The CPANN model consists of an input hidden layer called the Kohonen layer and an output layer called the Grossberg layer. The Kohonen networks are used to cluster the dataset into previously unknown groups. This model was applied to 1000 alpha helical peptides generated by modLAMP [179] de novo design engine to predict their anticancer property. The modLAMP package is a Python based software package that is used to design, classify and visually represent peptide data. The top 15 peptides along with three random peptides and three negative control peptides from the library were synthesized and tested in vitro on MCF7 and A549 cell lines. Out of the 15 positive peptides, six showed anticancer activities. Five out of these six showed activities against both cell lines, implying the multi-target prediction ability of this method.

Caceres-Delpiano et al. [175] developed a deep learning model that can design peptide sequences that are analogous in structure to a representative peptide. In this model, each amino acid was considered as a token and represented the protein as a vector. Their network architecture was made up of two Bi-LSTM layers, each having 1024 hidden units. The model was trained on nearly 21 million amino acid sequences taken from the Pfam database and 20000 sequences from the SCOP database. The model could reconstruct the protein sequence and minimize cross-entropy loss to predict various information from the sequence, including secondary structure, structural similarity, and contact maps. This method achieved 91.2% accuracy for classifying structures from the SCOP database. The authors then tested the application of this method to generation of sequences that can adopt a known structure and thus can perform a desired function. They chose chitin binding as their proof of concept model. The generation cycle started with a poly-Ala sequence. A mutator would create a library of sequences starting from this input sequence and the structural similarity of these sequences to the desired structure were calculated using their predictor. The best peptides were then used as the new input and this cycle was continued until a structural similarity score greater than 3.5 to the starting structure was attained. These predicted structures were then subjected to molecular dynamics simulations in the presence of a chitin surface to test their anti-fungal properties and interactions. These de novo designed peptides were expressed in vivo and their various biochemical properties were assayed. The authors showed that these peptides were able to replicate the antifungal properties of the wild type peptide, AC2-WT.

Capechhi et al. [176] have used ML for designing non hemolytic AMPs. Their models were trained using a combination of predictive and generative RNNs on training sequences obtained from the DBAASP database, which contained annotated sequences with information about their activities and hemolytic properties, if known. The model was further fine-tuned via transfer learning using active and nonhemolytic peptides against specific strains: *P. aeruginosa*/*A. Baumannii* and *S.aureus*, respectively. These fine-tuned models were sampled to generate sequences of new AMPs. The generated sequences were further classified by the RNN AMP activity classifier and the hemolytic classifier sequentially. 28 short peptides (less than fifteen residues) were synthesized and tested for their minimum inhibitory concentrations (MIC) against bacteria. A MIC value ≤16mg/mL was considered as a threshold of the peptide’s antimicrobial activity. It was seen that 64% of these peptides were active below this threshold, and out of these, 50% were both active and nonhemolytic.

## 5. Future Outlook

Design of functional peptides and protein fragments that bind to protein interfaces offer an exciting opportunity for understanding and inhibiting protein-protein interactions and developing new therapeutics. The design methods aimed at mimicking interaction of one protein with another span a variety of techniques from stabilization of naturally occurring binding partners to designing new sequences at the interface to advanced deep learning methods.

Methods that start from an existing natural motif have resulted in generation of many high affinity peptide binders, some leading to companies based on peptide-based binders, as well as huge advances in the field of peptide and protein design such as ncAA parametrization, cyclic peptide conformational sampling and prediction, and machine learning methods that can learn from sparse datasets. However, mimicking naturally occurring motifs inherently limits the design to cases where a structure is known. Additionally, the obtained affinities are highly dependent on the starting motif affinity and downstream mutagenesis and optimizations are often required to obtain peptides that outcompete the native binder.

The paradigm in recent years has shifted more towards application of automated methods and computational approaches for designing peptides and predicting their functions. Computational methods such as Rosetta-based design of peptides and MD simulations, while successful, are still hampered by the limitations of their underlying score function. Computational interface metrics often fail to fully justify the experimental results and predicting the binding mode is still an extremely challenging task. Improvements in the score function as well as more efficient sampling methods will improve the power of these methods in designing new functional peptides.

Learning-based methods for prediction and design of peptides are still at their early phases. The major roadblock in the application of these methods is lack of experimental data. To empower these approaches, we need both technological advances in learning methods and increased training data. To this end, approaches that combine high-throughput testing of peptide libraries with learning methods are of high value as they generate much needed labeled datasets for peptide functions. An example can be integration of computationally designed peptides with library-based screening methods such as phage display or mRNA display to generate a focused library whose screening can generate more informative results.

The huge leap in our ability to predict protein structures has inspired many researchers to develop novel approaches for predicting peptide structures, especially in complex with their binding partners. Access to accurate predictions of protein-protein or protein-peptide complexes combined with a more accurate prediction of binding affinities from these structural data will be transformational for the design of peptide binders, particularly in designing selective binders, an area that still lacks a general and robust pipeline.

Most peptide binders are designed with the ultimate goal of being used as a therapeutic. Binding is the first step in the long process of therapeutic development. Many high affinity candidates fail in the downstream steps due to undesired pharmacokinetic properties, half-life in the body, toxicity, and more. Additionally, most binding assays fail to test for cell permeability for intracellular targets, resulting in high affinity binders that cannot access the intracellular targets they’re designed to bind to due to lack of permeability. Thus, predicting permeability and pharmacokinetic properties will be an important area of focus for downstream applications. Lack of large-scale data for these features necessitates the use of new features and methods for prediction from noisy datasets with high failure rates.
